# Speech therapy in head and neck cancer

**DOI:** 10.1016/j.bjorl.2021.02.002

**Published:** 2021-02-13

**Authors:** Vaneli Colombo Rossi, Juliana Lopes de Moraes, Camila Ferreira Molento

**Affiliations:** a Universidade de Campinas, Departamento de Otorrinolaringologia e Cirurgia de Cabeça e Pescoço, Campinas, SP, Brazil; b Hospital Erasto Gaertner, Departamento de Cirurgia de Cabeça e Pescoço, Curitiba, PR, Brazil

Phono-oncology, or speech therapy in oncology rehabilitation, is an approach still not recognized as a specialty, but a reality in the practice of several oncological hospitals and in scientific research for over 20 years. Speech therapists with specific backgrounds and expertise in oncological rehabilitation act in this field. Within hospitals or clinic environments, these professionals work with a multidisciplinary team composed of surgeons, nurses, maxillofacial surgeons, physiotherapists, psychologists, and nutritionists who together assertively conduct the rehabilitation of patients ensuring maximum autonomy, social reintegration, and quality of life. The speech therapist is the instrument to provide the reestablishment of patient communication and feed safely, reinserting him in society as a communicator and adapting the feeding functions providing physical, emotional, and social well-being, significantly improving the quality of life.^1^, ^2^

Speech-language pathology occurs in the presence of head and neck tumors that affect the regions of the nose, lips, tongue, jaw, maxilla, hard and soft palate, nasopharynx, oropharynx, hypopharynx, larynx, thyroid, salivary glands, and oral cavity. Due to these tumors' location, the proposed treatments of surgery, chemoradiotherapy, or combination, will result in temporary or permanent speech disorders. The oncological speech therapist is responsible for coordinating the comprehensive care with the multidisciplinary team, both in primary care (prevention, promotion, support, palliative care) and in specialized care, following from the stage of diagnosis, before, during, and after clinical or surgical treatment.^1^

The treatment options for head and neck cancer are surgery, radiation therapy, chemoradiotherapy. It is common to implement combined protocols. Radiotherapy, the most common treatment in head and neck surgery, restricts cancer cells' reproductive potential, despite the advantage over surgery in terms of organ preservation, causes numerous local and generalized, temporary, or permanent adverse events. The speech therapist must identify and intervene in all stages of the radiotherapy process to minimize the sequelae related to swallowing and phonation that can severely impact life quality during treatment.

Unfortunately, all treatment options cause sequelae with a significant impact on the stomatognathic system's essential functions (suction, breathing, swallowing, speech, and chewing.) Complications resulting from cancer and its treatment can lead to mutilations and physiological changes, such as dysphagia, aspiration, chewing difficulties, changes in speech, and aesthetic changes that compromise these patients' physical and psychosocial aspects.^3^, ^4^

Ideal moment of speech therapist intervention begins with the diagnosis of cancer before the treatment. It is essential to provide guidance and clarifications to the patient’s family about the possible sequelae that may arise regardless of the treatment to be performed as well as to create a professional relationship that provides numerous benefits, greater adherence to treatment.^1^

However, in general, the speech therapist's performance, in most cases, is restricted to the healing phase. There are very few instances when the speech therapist participates during the pre-treatment phase in a multidisciplinary team and in the therapeutic decision. The most common is the performance after surgical treatment and or chemoradiotherapy. In surgical cases, speech therapy usually starts 15 days after surgery if there are no complications. This period varies according to each hospital and depends on the indication of the medical team. When allowed to start activities, the speech therapist performs a clinical and instrumental evaluation of all the remaining structures and functions.

In the evaluation, oral motricity, voice, swallowing, articulation pattern, feeding tube presence, provisional or permanent tracheostomy, and respiratory conditions are observed. When indicated, additional tests may be requested. The objective assessment is, through clinical reasoning, to identify the changes that have been made and the reason for such changes to define the diagnosis, prognosis, and the appropriate therapeutic approach.^4^

In the initial phase of treatment, speech therapy's primary objective in head and neck cancer is the rehabilitation of swallowing. Most of the time, the patient undergoing surgery uses a feeding tube, gastrostomy or jejunostomy, to eat. When the patient can swallow safely, without aspiration and with adequate oral transit time, the alternative feeding route can be removed. During the training for the oral route's reintroduction, the speech therapist defines the most appropriate consistency of the food and starts to perform sensory stimulation, maneuvers to protect the airways and postures, strengthening exercises followed by the function. A set of actions to facilitate swallowing is indicated for each patient.

Regarding vocal rehabilitation, a group of patients in particular that stands out is the total laryngectomies patients. The voice, the main instrument of identity, is directly affected in this type of surgery. With the removal of the laryngeal framework, the patient loses the ability to communicate vocally, becoming vocal disabled. For the reestablishment of communication, self-esteem, and social reinsertion, the speech therapist must act in the pre-surgical period by harboring, providing guidance, presenting the methods available for vocal rehabilitation as stoma care after surgery, using the appropriate supplies for this purpose.

Speech therapy monitoring contributes to expand the communicative potential, respecting the rehabilitative expectations and limits of the disease. Rehabilitation will seek to mitigate the anatomical changes detected in the patient, leading to improved quality of life and resocialization. This monitoring will be short, medium, or long term, depending on the sequelae's severity.^5^

However, the process does not always develop in this ideal way. In a retrospective descriptive study conducted in Olinda/PE in 2016, with a quantitative approach, cancers of the tongue and oropharynx were the main primary tumor sites found among the subjects who died. Furthermore, in this sample, only a few received an indication for speech therapy during the disease.^5^

Phononcology is busy dealing with diseases' consequences and, despite an little known subspecialty in the speech therapy field, relates specifically to a rehabilitation process among cancer patients with tremendous scientific research in the literature.

## Conflicts of interest

The authors declare no conflicts of interest.
